# The Importance of Suicide Risk Formulation in Schizophrenia

**DOI:** 10.3389/fpsyt.2021.779684

**Published:** 2021-12-16

**Authors:** Isabella Berardelli, Elena Rogante, Salvatore Sarubbi, Denise Erbuto, David Lester, Maurizio Pompili

**Affiliations:** ^1^Department of Neurosciences, Mental Health, and Sensory Organs, Faculty of Medicine and Psychology, Suicide Prevention Centre, Sant'Andrea Hospital, Sapienza University of Rome, Rome, Italy; ^2^Department of Psychology, Sapienza University of Rome, Rome, Italy; ^3^Department of Human Neurosciences, Sapienza University of Rome, Rome, Italy; ^4^Psychology Program, Stockton University, Galloway, NJ, United States

**Keywords:** suicide risk, schizophrenia, prevention strategies, risk formulation, risk assessment

## Abstract

Suicide is a cause of early mortality in nearly 5% of patients with schizophrenia, and 25–50% of patients with schizophrenia attempt suicide in their lifetime. Evidence points to numerous individual, clinical, social, and psychological risk factors for suicide in patients with schizophrenia. Although recognizing suicidal risk factors in schizophrenia is extremely important in suicidal risk assessment, we have recently witnessed a change in suicide risk management that shifts the focus from suicide risk assessment to suicide risk formulation. Suicide risk formulation is dependent on the data gathered in the suicide risk assessment and assigns a level of suicide risk that is indispensable for the choice of treatment and the management of patients with a high suicidal risk. In this article, we extend the suicide risk formulation model to patients with schizophrenia. Suicide risk formulation results from four different areas that help clinicians collect as much information as possible for the management of suicidal risk. The four distinct judgments comprise risk status (the risk relating to the specific group to which the patient belongs), risk state (the risk for the person compared with his baseline or another reference point in the course of his life), available resources (on whom the person can count during a crisis) and foreseeable events (which can exacerbate the crisis). In schizophrenia, the suicide risk formulation model allows the clinician to evaluate in depth the clinical context of the patient, the patient's own history and patient-specific opportunities for better choosing and applying suicide prevention strategies.

## Suicide Risk Formulation In Schizophrenia

Suicidal behavior in schizophrenia is an underestimated event, with 25–50% of these patients attempting suicide in their lifetime (1–3). It is also a frequent cause of early mortality, affecting nearly 5% of patients with schizophrenia (4, 5). Suicide may occur at any time during the clinical course of schizophrenia, although several studies have suggested that the highest suicide risk occurs during the first 10 years of illness (6, 7). Previous studies have reported numerous individual, clinical, social, and psychological risk factors for suicide in patients with schizophrenia (8, 9). Furthermore, schizophrenic patients who attempt suicide typically use lethal and violent methods requiring urgent medical attention and hospitalization (10).

Due to the importance of this outcome in patients with schizophrenia, suicide risk formulation and management are essential skills for clinical practice (11, 12). Many strategies can be used to prevent suicide, and awareness of suicide risk factors in patients with schizophrenia is necessary to manage suicide risk better (13).

Many factors associated with suicide in schizophrenia have been identified, but attempts to identify high-risk patients have so far produced too many false positives to be clinically useful. Suicide prevention strategies can be improved by assessing several suicide risk factors simultaneously. Although recognizing that suicidal risk factors in schizophrenia are extremely important, we have recently witnessed a change in suicide risk management that shifts the focus from suicide risk assessment to risk formulation. Suicide risk formulation is a process by which the clinician forms a judgment about the patient's suicide risk based on data collected during the suicide risk assessment, and suicide risk formulation would seem to be indispensable for treating and managing a suicidal crisis (14, 15). Pisani et al. (16) supported this paradigm shift, and they pointed to the necessity for rethinking the formulation of suicide risk from a predictive to a preventive perspective. The authors discussed that, usually in clinical practice, the assessment of suicide risk had been based on the expression “low,” “moderate,” or “high” risk, and they argued that this type of assessment had poor reliability and validity (16). The model proposed by those authors provides for a risk assessment that does not use a categorical approach. According to those authors, suicide risk formulation is based on four different areas that help clinicians collect as much information as possible to manage suicide risk. The four distinct judgments involved in suicide risk formulation comprise: risk status (the risk relating to the specific group to which the patient belongs), risk state (the risk of the person compared with his baseline or another reference point in the course of his life), available resources (on whom the person can count during a sudden crisis) and foreseeable events (which can exacerbate the crisis). This model redefines the concept of suicide risk formulation as a concise, empirically-based summary of a patient's immediate distress and resources at a specific moment and place (16).

In this article we extend the suicide risk formulation model to patients with schizophrenia. In schizophrenia, the suicide risk formulation model allows the clinician to evaluate in depth the clinical context of the patients, the patient's own history, and patient-specific opportunities for better application of suicide prevention strategies.

## Suicide Risk Status in Schizophrenia

The evaluation of the risk status compares suicide risk factors in a patient relative to other patients in a given population. For this reason, risk status is expressed using comparative forms such as “higher than,” “similar to,” or “lower than” in relation to a relevant comparison group. These factors tend to be more enduring (i.e., fixed, historical, and static), comprising patients demographic features. Other risk factors are mainly involved in the acute phase of schizophrenia and in the remission phase (16).

Demographic features, such as Caucasian race, male sex, younger age, and being unmarried, seem to be associated with increased suicide risk in patients with schizophrenia and are considered long-term status risk factors (17–20). Recently, Dickerson et al. (21) studied 733 patients with a schizophrenia spectrum disorder in order to examine the role of different variables in suicide risk. This study found that being Caucasian (vs. African American) and male was associated with higher hazard rates for suicide. Furthermore, a meta-analysis and meta-regression of 96 studies (22) confirmed that being male was consistently associated with suicide in patients with schizophrenia but a protective factor against suicidal attempts. The authors explained this result as consistent with the “gender paradox” phenomenon (23) in which females present higher rates of suicidal ideation and non-lethal behavior than do males. Suicide mortality is typically lower for females than for males, probably because women are more likely to use less lethal methods than do men. Regarding the relationship between age and suicidal risk in schizophrenia, several studies have demonstrated that younger patients have an increased likelihood of attempting suicide (24–26).

Furthermore, studies on marital status and suicide risk indicate that being married is a protective factor for suicidal risk, and married patients indeed have more family support than patients who are not married (27). Finally, the prevalence of suicide risk in patients with schizophrenia differs across countries (28). For example, several studies have demonstrated that patients with schizophrenia who live in high-income countries were more likely to attempt suicide than those in the low- and middle-income countries. Furthermore, patients who live in North America or Europe and Central Asia present a higher prevalence of suicide attempts than patients in South Asia, sub-Saharan Africa, East Asia, and Pacific areas (28). Differences in socio-cultural and economic contexts (such as societal discrimination and stigma) and the prevalence of substance abuse (such as alcohol and cocaine) may explain these differences (29–32).

Through the model of suicide risk formulation, previous suicide attempts are among the essential enduring risk status factors for suicidal behavior (33–35). Furthermore, repeated attempts exponentially increase the risk of a lethal act (36, 37). A multicenter randomized trial conducted by Fleischhacker et al. (the ZODIAC Study) (38) analyzed 18,254 patients with schizophrenia to understand better the features of completed and attempted suicide in this population. The results confirmed that previous suicide attempts were the strongest predictor of completed suicide and future suicide attempts. In addition, using logistic regression, Li et al. (39) examined 64 inpatients with schizophrenia and found that previous suicide attempts were an independent risk factor for suicide and useful for better suicide risk formulation.

Other enduring suicide risk factors that play an important role in suicide risk formulation include adverse childhood experiences (ACEs). It has been postulated that ACEs are nearly three times more frequent in patients with schizophrenia and other psychoses than in the general population (40). Adverse childhood experiences that include physical, sexual and emotional abuse, as well as neglect, are present in about 30% of the general population and can impair the individual's sense of security (e.g., substance-abusing parents, divorce, incarceration of a family member, etc.) (41). Is well-known that ACEs increase the risk of mental health consequences in adulthood, including depression, anxiety, psychosis, post-traumatic stress disorders, dissociative disorders, and personality disorders (42). In patients with schizophrenia, ACEs have been associated with the development of psychotic episodes (43), an earlier onset of schizophrenia (43), a higher incidence of positive and negative symptoms (44–49) and a higher risk of suicidal behavior (43, 45, 46, 50, 51). Several studies have proposed that the risk of developing psychosis is higher in patients exposed to multiple ACEs. A possible explanation is that repeated exposure to adverse experiences increases mesolimbic dopamine reactivity, causing delusions and hallucinations (40, 52–56). Several studies have reported an association between negative childhood experiences and suicidal risk in psychiatric patients (57–59). Consistent with these findings, Hassan et al. found that patients with schizophrenia who had experienced ACEs were more likely to attempt suicide than patients who did not report ACEs. Moreover, recently Prokopez et al. (60) studied 100 patients with schizophrenia and observed that multiple ACEs were associated with a higher frequency of suicide attempts. Notably, the authors observed that men and women with various adverse experiences during childhood reported more suicidal ideation and a higher frequency of at least one suicidal attempt. The women with multiple ACEs had a higher number of suicide attempts.

Family functioning, that is, the ability to adapt oneself to changes determined by the levels of cohesion, flexibility, and communication within a family, is often compromised in families of individuals with schizophrenia and is an important enduring factor in suicide risk formulation (61–63). Moreover, it seems that disrupted relationships with family members may increase suicide risk (64–67). For example, in a sample of 263 patients with schizophrenia spectrum disorders, Chang et al. (68) found that suicidal behavior was associated with poorer family relationships. In addition, a study by Demir (69), that analyzed 350 psychiatric outpatients, found a significant association between suicidal behavior and abnormal emotional expression by a family member who exhibited excessive, hostile, and judgmental emotions toward the psychiatric patient.

Among other factors involved in suicide risk status, premorbid functioning in psychotic disorders influences clinical and functional outcomes (70). For example, in a study by Pompili et al. (71), good premorbid functioning increased the risk of completing suicide. The authors explained this result as being consistent with the “demoralization syndrome” (72), in which a good level of functioning and insight into the illness may cause hopeless feelings and suicidal thoughts (73). Conversely, Bakst et al. (74), who assessed premorbid functioning in a sample of 444 individuals with a first psychotic episode, found that a higher likelihood of suicide attempts before the first psychiatric hospitalization was associated with poorer premorbid functioning. Nevertheless, when the sample was divided into two groups (schizophrenia vs. other psychoses), the association was no longer significant in the patients with schizophrenia. In contrast, the association remained significant in the patients with other psychoses. The authors interpreted this result by taking into account the heterogeneous phenomenology of schizophrenia.

Different clinical features associated with schizophrenia can be enduring suicide status risk factors. The age of schizophrenia onset has a crucial role in understanding the developmental and distinctive features (i.e., genetic and environmental antecedents) of the disorder that appear in early adulthood and become chronic and impairing (75). Early-onset schizophrenia is associated with a poorer prognosis and worse psychological, social and biological outcomes (76, 77). In particular, suicide risk seems to be associated with an early onset of schizophrenia, as confirmed by Vinokur et al. (78) in a study of 138 patients with schizophrenia. Moreover, in a British follow-up investigation (79), most suicides occurred within the first 2 years of the onset of the psychotic disorder. A study by Castelein et al. (80) that followed 424 patients with schizophrenia for 20 years found that the percentage of deaths that was from suicide declined over time from 11.0 to 2.4%. Thus, although higher suicide rates have been identified in younger patients, suicide risk remains stable throughout life in individuals with schizophrenia (2). Several authors also reported that a longer illness duration was significantly associated with lifetime suicide attempts (81, 82). Recently, Chang et al. (68) showed that a longer illness duration was one predictor of lifetime suicide attempts in a sample of 263 patients with schizophrenia. Dai et al. (83) replicated this result by comparing 908 patients with schizophrenia with suicide attempts to those without suicide.

Status factors involved in the suicide risk formulation in the acute phase of schizophrenia include the type of psychotic symptoms and categorization of schizophrenia, depressive symptoms and hopelessness, alcohol abuse, anxiety symptoms, insomnia, and illness insight. Evidence regarding the predictive value of positive and negative symptoms on suicide risk is still conflicting (80, 84, 85). Andriopoulos et al. (86) found that both positive and negative symptoms were increased in individuals with suicide ideation (vs. those without suicide ideation). Gill et al. (87) examined 42 individuals at high risk for psychosis and found a significant association between suicide ideation and negative symptoms.

However, the association between specific subtypes of schizophrenia and suicide risk is still controversial. The review conducted by Pompili et al. (33) found no significant differences in suicide risk according to the classical categorization of schizophrenia as catatonic, hebephrenic, or undifferentiated. On the other hand, paranoid schizophrenia seems associated with a higher suicide risk, probably as a result of the later age at onset (88). Possible explanations for the increased risk of suicide associated with increased age at onset of illness in patients with paranoid schizophrenia might include the stress these patients face, the deterioration in cognitive functioning, and having had a family and occupation during their early adult years (36, 89). Furthermore, a relation between command hallucinations and suicide risk has been postulated, but the data are still controversial. Fenton et al. (89), in a long-term follow-up study, observed that patients with schizophrenia who died by suicide presented lower negative symptom severity upon admission and more often had two positive symptoms (suspiciousness and delusions) than patients without suicidal behaviors (89). Repeated psychiatric hospitalizations are a common feature of patients with schizophrenia due to the disease's chronic nature and poor medication adherence (90).

Moreover, re-hospitalization seems to be strongly associated with suicidal ideation and behaviors, as Fleischhacker et al. (38) highlighted in their study of potential baseline risk factors for attempted suicide in 18,154 patients with schizophrenia. The results showed that a history of more than five hospitalizations was, alongside previous suicide attempts, the most substantial variable associated with attempted suicide. Zhang et al. (91) assessed 520 inpatients with schizophrenia and compared suicide attempters and non-attempters. They found that patients who had attempted suicide reported significantly more frequent hospitalizations, and a logistic regression analysis confirmed that suicide attempts were associated with more hospitalizations.

An additional factor in evaluating suicide risk status in schizophrenia is patient insight into the illness. Most researchers have defined insight as being comprised of at least three domains: awareness of the disease, awareness of the need for treatment, and awareness of the consequences of the disorder (92). Some studies (93, 94) have demonstrated that hopeless awareness of the severity of the disorder (schizophrenia) was one of the most important predictors of completed suicide in patients with psychotic disorders. However, it is still uncertain whether insight was directly related to suicide or mediated by its influence on hopelessness. Several studies have demonstrated that illness awareness is associated with increased suicide risk in patients with schizophrenia (95–97). In a recent study of 100 psychiatric inpatients, Berardelli et al. (98) reported that patients with higher scores on the insight-high dimension had a 1.35 greater odds of having a higher suicide risk, indicating that greater illness insight is involved in suicide risk.

Current depressive disorders were strongly associated with suicide in patients diagnosed with schizophrenia (99, 100). More than 50% of patients who died by suicide had symptoms of depression at the time of the suicide (8), and it has been suggested that a depressive disorder may trigger suicidal behavior in vulnerable patients with schizophrenia (36). In addition, *hopelessness* is an important risk factor in people diagnosed with schizophrenia (101), even in the absence of a concomitant depression (94). Several studies have also reported that symptoms of demoralization in patients with schizophrenia are related to suicide risk (73). Depressive symptoms occur in different phases of the psychosis, including prodromal, acute, and post-psychotic phases (102–104). It has been proposed that depressive dimensions are intrinsic to schizophrenia psychopathology, in positive, negative, and disorganized symptom clusters (104). The close linkage between psychotic symptoms and depression, especially in the prodromal phase, suggested that depression in schizophrenia may be the severe end of a dimension of affective dysregulation from adolescence progressing to the early stages of psychosis as the illness crystalizes (105). Furthermore, depression could be a psychological reaction to the diagnosis of schizophrenia and its implications for patients' lives or could be related to early risk factors such as a childhood trauma (103). Addressing psychotic depression is important not only in suicide formulation but also for schizophrenia management as it is related to relapse, greater substance-related problems, poorer life satisfaction, mental functioning, family relationships, and medication adherence (106).

Substance abuse is a common phenomenon among individuals with schizophrenia (107). Up to 50% of patients with schizophrenia exhibit either alcohol or illicit drug dependence, and more than 70% are nicotine dependent (108). In particular, heavy cannabis abuse has been reported to be a stressor, eliciting relapse in patients with schizophrenia and related disorders (109). Although it is difficult to compare the relative impact of different mental health problems with suicide risk, alcohol and drug use disorders have been strongly linked to suicide risk (110, 111). Multiple potential links, including genetic vulnerability, treatment side effects, and psychosocial factors, have been discussed as possible pathways (112). One explanation for the increased incidence of substance use in patients with schizophrenia is the self-medication hypothesis (113). Individuals with a substance use disorder (i.e., a diagnosis of either alcohol or drug abuse or dependence) are almost six times more likely to report a lifetime suicide attempt than those without a substance use disorder (114). In addition, evidence has suggested that alcohol abuse is a predictor of suicide (7, 115). However, some authors have suggested that alcohol abuse may be associated with suicide attempts but not with completed suicide (116). As for drug abuse, most studies have reported an association with increased suicide risk (117) and impulsiveness (118). In particular, abuse of stimulants (cocaine, amphetamine) increase the risk of attempted suicide (119).

Anxiety symptoms are highly prevalent in schizophrenia and occur in up to 65% of patients (120, 121). Anxiety symptoms are strongly associated with depressive symptoms, somatization, and feelings of guilt in patients with schizophrenia (122, 123). Panic attacks have also been associated with suicide risk in schizophrenia (120). However, although anxiety symptoms do not discriminate suicide ideators from attempters, anxiety symptoms significantly predict general suicidality in schizophrenia (124). Patients with schizophrenia and anxiety symptoms have a lower quality of life but higher insight into their illness than those without anxiety symptoms (124).

In a case-control study, Pompili et al. (125) suggested the role of insomnia as a suicide risk factor. Previous research on the relationship between suicide and sleep disturbances has noted that those patients with schizophrenia who exhibited suicidal behavior presented increased overall rapid eye movement (REM) activity (126).

Other schizophrenia related factors, mainly present in the remission phase, are involved in suicide risk status including loss of confidence in pharmacotherapy, fear of further mental breakdown and fear of acute symptomatology.

Finally, risk status is the assessment of different enduring risk factors based on the clinical context and patient population, on the patient's history, and on patient-specific opportunities for prevention.

## Suicide Risk State in Schizophrenia

Suicide risk refers to a person's current risk compared with his/her own risk at baseline or at another set point in time. Factors involved in risk state are more dynamic and related to the moment-to-moment clinical status of patients. Together, risk status and risk state allow clinicians to understand the patient's current vulnerability among their population, context, and time. The risk state formulation focuses on temporal changes and on the effect of the distress on the patient's life. The state risk factors that are mainly involved in the acute phase of schizophrenia include the presence of suicide ideation or recent suicide behavior, recent loss, social isolation, new hospitalizations, loss of faith in treatment, excessive treatment dependence, awareness of the illness, and social alienation (16).

The presence of suicidal ideation during the acute phase of schizophrenia must be carefully evaluated during the clinical interview. With the aid of appropriate psychometric tools, recent stressors and precipitant events represent important dynamic factors capable of modifying the suicide risk state. Among the various stressors, recent loss is often considered a state risk factor for suicide in patients with schizophrenia (1, 22, 99). Gallego et al. (127) investigated 3,322 patients diagnosed with schizophrenia and affective disorders and reported a significant association between recent financial or relational loss and current suicide attempts. In a sample of 180 patients with first-episode psychosis, Fedyszyn et al. (128) demonstrated that one of the most vital risk factors for suicide was a recent negative event, such as a traumatic or stressful experience. Social isolation, which consists of disrupted or non-existent interpersonal contacts and relationships, is a well-known suicide risk factor in schizophrenia (129–131). Recently, several papers have further addressed this relationship. For example, Bornheimer et al. (132) investigated the relationship between social isolation, psychosis, and suicide ideation and found a mediating effect of social isolation in the relationship between psychosis and suicide ideation. In addition, the authors identified an indirect path between positive symptoms (hallucinations and delusions) and suicide ideation through social isolation. Conversely, several studies have shown the protective role of social support in reducing suicide risk (133–135). Social support increases feelings of belongingness and prevents negative appraisals after stressful events, and the presence of individuals can physically prevent suicide attempts. However, only a few studies have analyzed the role of poor social support in suicide risk in schizophrenia spectrum disorders. Xie et al. (50) reported a significant negative correlation between social support and suicide ideation in a sample of patients with schizophrenia. In a study of 212 patients with schizophrenia, Pješčić et al. (136) found a higher prevalence of poor social support and social isolation in patients with suicide ideation.

The state risk formulation also evaluates several features related to schizophrenia and its treatment during the acute and remission phases of the illness, including recent discharge from a psychiatric ward and fear of a new hospitalization. Current discharge from a psychiatric ward as a risk factor for suicide was noted in a recent study (137) that reported a suicide rate of 178 per 100,000 person-per year within the first 3 months after discharge. Moreover, the lack of adequate outpatient healthcare seems to increase suicide risk (137). Erlangsen et al. (138) assessed 248 suicides in Denmark between 1990 and 2006 and identified recent discharge as a significant risk factor for suicide. Waiser et al. (139) examined 2,881 patients with schizophrenia through a survival analysis method. They found that ~32% of suicides occurred within 6 months after psychiatric hospitalization, with the rate rising to 48% within 1 year. Lastly, Lopez-Morinigo et al. (140) analyzed 426 suicides (71 patients with schizophrenia spectrum disorder and 355 controls) and demonstrated a significant association between recent hospital discharge and completed suicide in the schizophrenia group. In a systematic review by Hawton et al. (8), agitation or motor restlessness seemed to be associated with suicide risk in patients with schizophrenia during the acute phase of the illness.

It is well-known that schizophrenia can modify many facets of patients' experience, including language, emotion and intersubjectivity, all factors related to social alienation. Therefore, these symptoms of schizophrenia should be understood as human psychopathological phenomena and not only as sub-products of a malfunctioning brain (141). Disturbances in both language, emotion and social interactions in patients with schizophrenia have received some research attention. However, little attention has been paid to these dimensions' subjective experience, particularly the personal meaning for patients with schizophrenia (142). Their language, emotion and encounters with other people has been poorly investigated. Furthermore, hyper-reflexivity, which refers to a type of intensified self-consciousness, diminished self-affection that represents a decline in consciousness of oneself as the subject of experience, and alteration of interactions with the world are other important dimensions involved in a loss of vital contact with reality in patients with schizophrenia (143). Schizophrenia should be viewed as a disorder of the person and not only of the brain. We can understand the importance of a suicide risk formulation based on the evaluation of subjective dimensions and external-social dimensions that are strongly connected to suicide risk (16).

In the acute phase of the illness, patients with schizophrenia may voluntarily decide to enter in a hospital if their symptoms are severe and painful. Awareness of the severity of psychotic symptoms and the fear of a new hospitalization can affect the patient's mental state, increasing hopelessness, helplessness and social isolation, all risk factors for suicide. Furthermore, clinicians can decide to commit a patient involuntarily. The decision to involuntarily commit an individual has become more challenging due to rapid changes in health care. It is important to remember that patients who are forced into treatment may develop a sense of distrust toward treatment providers and family members, which can delay recovery in the long run and can increase suicide risk. Alongside the increased awareness of symptoms, fear of the ineffectiveness of drug therapies and fear of becoming dependent on the various treatments can also increase the risk of suicide of patients with schizophrenia (144).

Among the various acute symptoms of schizophrenia, agitation seems to be involved in suicide risk. Pompili et al. (125) conducted a retrospective case-control study comparing 20 patients with schizophrenia who died by suicide with 20 living controls and found that agitation and motor restlessness was much more common in the suicides Furthermore, the guidelines drafted by the European Psychiatric Association (145) highlight the role of agitation in affecting suicidal behavior in patients with schizophrenia. Suicide risk also seems to be associated with poor adherence to treatment as shown in several studies (8, 146). For example, Hering and Erkens (147) analyzed 603 patients with schizophrenia. They observed a four-fold increased risk of suicide in the group that interrupted treatment with antipsychotic drugs compared with the group that continued treatment. In addition, Novick et al. (148) reported that, of the 6,731 patients in the study, 28.8% of the sample was non-adherent to treatment, and this feature was associated with suicide attempts. Consistent with this, Ward et al. (149) analyzed 3,291 patients with schizophrenia, and their results demonstrated that good adherence was a protective factor against suicide.

Antipsychotic medications are known to be associated with several movement disorders, acute side effects, late-onset side effects, tardive dyskinesia, and extrapyramidal symptoms (EPSs). The latter include acute dystonia and parkinsonism which can be present also in the remission phase of schizophrenia (150). In a double-blind, randomized controlled multicenter trial on 298 patients with first-episode schizophrenia who were in treatment with risperidone or haloperidol, suicide ideation was associated with akathisia, suggesting that EPSs may have a promoting effect in suicide risk (151). However, the findings of Reutfors et al. (150) demonstrated a lower suicide risk in patients with a history of EPSs. The authors explained their results by noting that these patients were characterized by better adherence to treatment, higher dosages, and a prevalence of polypharmacy.

Other suicide state factors include the presence of medical disorders and poor mental health conditions (152). Patients with schizophrenia present higher mortality rates, probably due to the presence of a medical disorder and unhealthy lifestyle behaviors, in addition to psychiatric impairment and psychotropic medication use. The presence of physical comorbidity, the need for new pharmacological treatments and specialist visits, and fear of the prognosis and for the quality of life could increase the psychological fragility of patients, thereby becoming state risk factors.

In conclusion, the factors involved in risk state are dynamic and malleable and relate more to moment-to-moment clinical status in the acute phase of schizophrenia. Together, risk status and risk state allow clinicians to understand better an individual's current vulnerability in this population, context, and time.

## Available Resources and Foreseeable Changes in Schizophrenia

A suicide risk formulation model based only on a categorical label for suicide risk state requires detailed additional information in order to plan risk prevention and the management of the patient. A complete suicide risk formulation, in combination with estimates of risk state and risk status, permits an assessment of the patient's available resources and future foreseeable changes (16).

Available resources are defined as resources immediately accessible to the patient and clinicians to support them during suicide crises in the acute phase of the illness. Recognizing the available resources for patients is different from assessing suicide protective factors, which often refer to general and epidemiologic factors known to decrease suicide risk across populations and are not immediately available during an acute suicidal crisis. In this sense, clinicians have to focus on the personal variables of each patient (coping strategies, resilience) and relational variables such as social support available during the acute phase of the crisis (16). Furthermore, when applying this model to patients with schizophrenia, the clinician, when identifying available resources, must consider the presence of psychotic symptomatology, patient insight, and factors that reduce collaboration between the patient and the resources.

Foreseeable changes are stressors that can increase or decrease suicide risk in the acute phase of schizophrenia. However, not all stressors increase suicide risk. Therefore, in the formulation of suicide risk, it is important to understand what makes an ordinary stressor a trigger for suicide (153). To better investigate the role of the various stressors, clinicians should investigate the subjective meaning or consequence of the stressor for the patient. In schizophrenia, foreseeable changes comprise the presence of substance use, school disciplinary action, inpatient discharge and social and relationship difficulties.

Furthermore, return to a conflictual environment after hospitalization and changes in pharmacotherapy are other foreseeable changes that need to be assessed. To better understand suicide risk in schizophrenia, it is also important to assess whether symptoms are increasing or decreasing and the meaning of these changes for the patient. The assessment of anger, impulsivity, isolation, depression, demoralization, and hopelessness is necessary for immediately implementing suicidal risk prevention strategies. Finally, the level of engagement between the clinician and the patient, the relationship with the clinician, and the degree to which the patient's report has been honest, reliable, and credible are other essential factors (153) (Table 1).

**Table 1:** Suicide risk formulation in schizophrenia.

**Suicide risk status**	**Suicide risk state**	**Available resources**	**Foreseeable changes**
• Socio-demographic features	•Comorbid psychiatric disorders	•Coping strategies	•Subjective meaning of the stressor
•Previous suicide attempts	•Insomnia	•Resilience	•Presence of substance abuse
•Adverse childhood experiences	•Recent losses	•Social support	•School disciplinary actions
•Family conflicts	•Social isolation	•Insight	•Inpatient discharge
•Premorbid functioning	•Recent discharge from a psychiatric ward	•Absence of psychotic symptomatology	•Social difficulties
•Early onset schizophrenia	•Agitation/motor restlessness	•Good collaboration with the clinician	•Conflictual environment
•Repeated psychiatric hospitalizations	•Poor compliance		•Changes in pharmacotherapy
•Higher insight levels	•Extra-pyramidal symptoms		
•Positive/negative symptoms			
•Longer duration of illness			

## Suicide Risk Formulation and Suicide Prevention Strategies in Schizophrenia

Suicide prevention in patients with schizophrenia is a complex phenomenon. Inadequate knowledge of suicide risk factors in patients with schizophrenia negatively affects the ability of clinicians to recognize patients at risk for suicide. Clinicians need to have better knowledge of suicide-related knowledge and suicide risk formulation in order to identify patients with high suicide risk. Careful assessment and management of psychotic symptoms, comorbid depression, hopelessness, demoralization symptoms and substance use disorders are also necessary to prevent suicide in patients with schizophrenia.

Several pharmacological and non-pharmacological strategies are available to reduce suicide risk in patients with schizophrenia. Undoubtedly enhancing adherence with medications is essential for alleviating psychotic and non-psychotic symptoms in schizophrenia. Patient-related features involved in the adherence with medications include demographic characteristics, newly starting treatment, younger age at onset of illness, alcohol dependence and other illicit substance use, homelessness, low levels of involvement in social activities, independent housing, and financial constraints (154). Lack of family support for adherence, or having no family, further contribute to non-adherence. Significantly higher IQs, executive functioning, memory, and verbal learning/fluency are also factors involved in medication adherence (155, 156). Medication non-adherence is associated with an increased risk for relapse of psychosis, persistent symptoms, and suicide attempts (157), making it indispensable to enhance adherence with medications.

Studies suggest that antipsychotic medications, including clozapine, risperidone, olanzapine and quetiapine, may reduce mortality and suicide risk in schizophrenia (158, 159). In 2002, the U.S. Food and Drug Administration (FDA) approved clozapine for decreasing suicide risk in patients with schizophrenia. Studies have suggested that atypical antipsychotic may be more effective than the use of typical antipsychotics (146, 160). Evidence for the ability of clozapine therapy to reduce suicidal behaviors has been highlighted in several studies since the late 1990s. Meltzer and Okayli (161) observed 88 neuroleptic-resistant patients treated with clozapine for 0.5–7.0 years and reported decreased suicidal behavior. Walker and Diforio (162) noted that suicidal behavior decreased in patients with current clozapine treatment compared with past users. Reinstein et al. (163), in a retrospective study of 295 patients, confirmed that clozapine treatment reduced suicide risk during continuous drug administration. More recently, Meltzer et al. (164), in a multicenter, randomized, international, 2-year study comparing the risk for suicidal behavior in patients treated with clozapine *vs*. olanzapine, observed that suicidal behavior was significantly less frequent in patients treated with clozapine. Several studies conducted by Tiihonen et al., confirmed that antipsychotic medication in general, particularly clozapine, was associated with lower suicide risk (165, 166). Furthermore, the same author observed that long-term treatment with antipsychotic drugs is associated with lower mortality, and clozapine seems to produce substantially lower mortality than any other antipsychotics (166). However, some antipsychotics, including clozapine and haloperidol and other antipsychotics, may increase the risk of depression (167). Tiihonen et al. (168) investigated whether using benzodiazepines, antidepressants, or multiple concomitant antipsychotics was associated with mortality in patients with schizophrenia. Their results demonstrated that the use of 2 or more antipsychotics was not associated with increased mortality. In contrast, antidepressant use was not associated with a higher risk for mortality and was associated with markedly fewer suicides. However, benzodiazepine use was associated with an increased risk of suicidal and non-suicidal deaths. Haukka et al. (169) observed that olanzapine, and to some degree clozapine, tended to perform well for suicide risk; which is in line with a review on the potential anti-suicidal effects of atypical antipsychotic (167). Taipale et al. (170) investigated the risk of attempted and completed suicide in patients with schizophrenia. The authors confirmed that clozapine is the only antipsychotic associated with a decreased risk of suicide. Several studies have also demonstrated that olanzapine, an atypical antipsychotic similar to clozapine, could effectively reduce depression and suicide risk in patients with schizophrenia (171, 172).

A study of 339 patients with psychotic disorders found that both olanzapine and risperidone were effective for the treatment of psychotic symptoms. However, only olanzapine produced a seven-fold lower risk of suicidal behaviors (173). In addition, olanzapine demonstrated a more significant anti-suicidal effect than haloperidol (174) and was similar to risperidone (175). Furthermore, quetiapine has been shown some potential effects for reducing suicidal risk, not only in patients with schizophrenia (167). Evidence about other atypical antipsychotic drugs on suicidal risk remains very limited. Whether all specific antipsychotics effectively prevent completed suicides also remains unclear. Long-Acting Injectable Antipsychotics (LAIs) have several advantages in terms of efficacy, safety and tolerability in treating schizophrenia. A better understanding of whether LAI treatment may decrease suicide risk by indirectly acting on a range of risk factors for suicide specific to patients with schizophrenia is of significant clinical importance. Pompili (176) suggested that long-acting injections of second-generation antipsychotics can be an effective treatment strategy to improve medication adherence and prevent suicide risk.

Furthermore, in addition to antipsychotics, other psychotropic medications, such as antidepressants and mood stabilizers, are often used in for patients with schizophrenia (177). Research suggests that the use of antidepressants together with antipsychotic drugs has been associated with a decrease in all-cause mortality, including suicide (169). However, Tiihonen et al. (165), in a large cohort study, observed that antidepressant treatment increases the risk of suicide attempts but not of completed suicides and death. In addition, a cohort study noted that antidepressant use in schizophrenia, compared with no use, was associated with a significant reduction in risk of completed suicide (166). However, two systematic reviews of randomized controlled trials confirmed the increased risk of self-harm or suicidal attempts related to selective serotonin uptake inhibitors (SSRIs) (160, 178).

Mood stabilizers have been used to augment the effects of antipsychotic drugs in patients with schizophrenia. Mood stabilizer medications may also effectively reduce depression, aggression and impulsivity in patients with schizophrenia when administered with antipsychotic therapy. Together with clozapine, lithium is the only drug showing anti-suicidal properties in bipolar and major affective disorders (171). A comprehensive meta-analysis of the effect of lithium in reducing suicide risk reported that both completed and attempted suicide were reduced by nearly five-fold, or 80% (179). Recently, several studies have suggested that lithium treatment reduces suicide attempts, suicides, hospitalization for suicide attempts, and other suicide spectrum disorders, compared with patients treated with other mood stabilizers (180–182). Although the beneficial effects of lithium on suicide risk in affective disorders are well-documented (183), data on whether this extends to patients with schizophrenia or schizoaffective disorder are lacking (184). The potential of other mood-stabilizing agents, such as lamotrigine, which has established antidepressant activity, is less well-understood than that of lithium, valproate or carbamazepine.

Non-pharmacological strategies are also crucial in the management of suicide risk in patients with schizophrenia. Psychosocial interventions, reality-orientated therapies, cognitive-behavioral therapy, cognitive remediation, supportive therapy, education and family intervention can be used together with pharmacological therapies in order to reduce suicide risk in patients with schizophrenia. Interventions such as vocational rehabilitation, social skills training, and supportive employment may also reduce social isolation and feelings of hopelessness, decreasing suicidality.

A few studies have examined the impact of specific psychotherapeutic and psychosocial interventions on suicide risk in patients with schizophrenia (36). Evidence suggests that supportive psychotherapeutic interventions that discuss acute symptoms, depression and hopelessness, daily difficulties, medications, adverse effects, social isolation, and stigma are necessary non-pharmacological suicide prevention strategies in patients with schizophrenia (185). Findings of a recent meta-analysis that examined 11 studies showed a statistically significant treatment effect of psychosocial interventions for suicide spectrum disorders individuals with psychosis (132).

Reviews and meta-analyses of cognitive behavior therapy (CBT) for psychosis have reported positive results for various symptoms (186, 187). A randomized controlled trial of cognitive behavioral therapy (CBT) for 90 patients with schizophrenia found that cognitive behavioral therapy was related to a significant reduction in suicidal ideation at the end of the psychotherapy and 9 months after the therapy (188). Additionally, psychodynamic treatments have been proposed to reduce suicide risk in patients with schizophrenia (189). Overall, clinicians should consider a phenomenological approach when treating suicidal individuals, pointing to the inner experience of the wish to be dead of each unique individual, avoiding the limitation of treating only single diagnostic entities (190, 191).

## Conclusions

Suicide in schizophrenia is a complex phenomenon that represents an ongoing challenge in clinical practice. This overview shows that suicide risk in schizophrenia is influenced by a variety of demographic, clinical, psychological, social, cultural, and environmental factors. However, identifying high-risk patients only with simple techniques of suicide assessment has so far produced too many false-positive results to be clinically helpful.

Suicide prevention strategies can be improved by simultaneously assessing and examining multiple risk factors and applying the suicide risk formulation model. Therefore, therefore, the aim of the suicide risk formulation model in schizophrenia is not the prediction but rather the formulation of a complete picture of the person. Promoting communication and collaboration between professionals, patients, and families should reduce suicide risk in the short term and in the long term. Structuring a targeted intervention plan that includes pharmacological and non-pharmacological strategies seems indispensable for managing suicide risk in patients with schizophrenia.

For psychiatrists and other clinicians working with patients with schizophrenia, arriving at a clear formulation of a patient's level of risk, based on a synthesis of clinical and non-clinical information, is a core competency for assessing and managing suicide risk. In addition, in clinical settings, the suicide formulation model allows for the prevention of future suicidal behavior and leading to practical safety and crisis response plans, which are the main objective of suicide prevention.

## Author Contributions

MP and IB: conceptualization. MP, IB, and ER: methodology. IB, DE, SS, and ER: data curation. IB and ER: writing-original draft preparation. DL and MP: writing, review, and editing. All authors contributed to the article and approved the submitted version.

## Conflict of Interest

The authors declare that the research was conducted in the absence of any commercial or financial relationships that could be construed as a potential conflict of interest.

## Publisher's Note

All claims expressed in this article are solely those of the authors and do not necessarily represent those of their affiliated organizations, or those of the publisher, the editors and the reviewers. Any product that may be evaluated in this article, or claim that may be made by its manufacturer, is not guaranteed or endorsed by the publisher.
